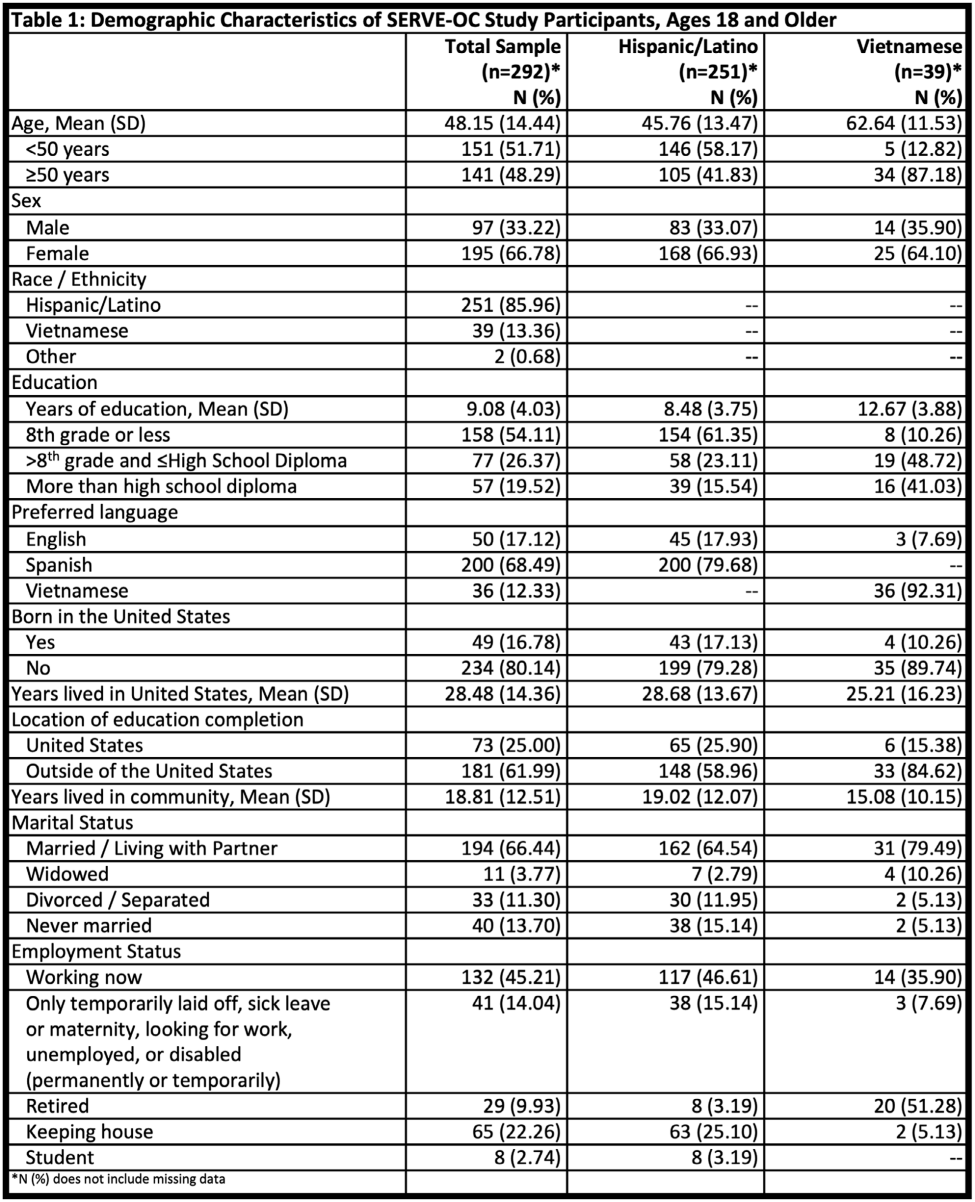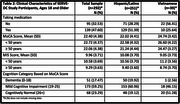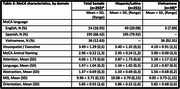# Exploring Education and Cognition in Minority Communities in Southern California: Insights from the SERVE OC Randomized Trial

**DOI:** 10.1002/alz70860_099210

**Published:** 2025-12-23

**Authors:** Alissa Kurzman, Bruce Albala, Xueting Ding, Megan Castro, Aryanna Chavez, Jeffrey Wing, Desiree Gutierrez, Darnisha Draughter, Cassandra Cardenas, Julie Huynh, Joceline Porron, Stephania Tovar Vargas, Bernadette Boden‐Albala

**Affiliations:** ^1^ Joe C Wen School of Population & Public Health, Henry and Susan Samueli College of of Health Sciences, University of California, Irvine, Irvine, CA, USA; ^2^ School of Medicine, University of California, Irvine, Irvine, CA, USA; ^3^ School of Pharmacy and Pharmaceutical Sciences, University of California, Irvine, Irvine, CA, USA; ^4^ Ohio State University, Columbus, OH, USA

## Abstract

**Background:**

Racial and ethnic minorities in the U.S. are disproportionately impacted by Alzheimer's disease and related dementias (ADRDs). Among U.S. Latinos, 11.1% are living with mild cognitive impairment (MCI) and 5.3% with mild dementia due to ADRD. Vietnamese Americans, one of the fastest‐growing ethnic groups in the country are also at risk of ADRDs as their population ages. Now, more than ever, timely and accurate ADRD screening is necessary for early intervention.

Cognitive assessments, including the Montreal Cognitive Assessment (MoCA), are vital for early screening of cognitive decline. However, performance may be influenced by educational attainment or cultural factors, leading to challenges in accurately differentiating cognitively impaired individuals from individuals with low educational status. This study examines the relationship between education and cognitive performance (MoCA total and domain scores) in Latino and Vietnamese participants of the community‐based, Skills‐Based Educational strategies for Reduction of Vascular Events in Orange County, California (SERVE OC) trial, to identify the domains most impacted by education.

**Methods:**

Data from the SERVE OC study were used (NIMHD P50MD017366). Study participants were restricted to randomized Latino and Vietnamese individuals, ≥18 years who completed the MoCA at baseline (December 2022‐April 2024) (*n* = 292). Multivariable regression models examined associations between education (≤8 years vs. >8 years) and MoCA total and domain scores adjusting for demographic and health factors and comparing findings across race/ethnicity.

**Results:**

Participants averaged 48.15(SD:14.44) years old (Latino: 45.76; Vietnamese: 62.64), were 66.78% female and 54.11% had ≤8 years of education. MoCA scores averaged 22.40;SD=4.38 (Latino: 22.02, SD=4.41; Vietnamese: 24.74;SD=3.42). Having ≤8 years of education was significantly associated with lower MoCA total scores (cBeta=‐2.79, *p* <0.01; aBeta=‐1.71, *p* <0.01) and for Latinos, lower scores in visuospatial/executive function (B=‐0.61;p<0.01), attention (B=‐0.86;p<0.01), and language (B=‐0.57;p<0.01) domains. Vietnamese participants with ≤8 years of education had lower abstraction domain scores (B=‐0.85;p<0.05). Mean MoCA scores 2.72 points lower in Latino participants compared to Vietnamese participants.

**Conclusion:**

Study findings underscore the importance of developing, validating and utilizing culturally appropriate and comprehensive cognitive assessments that transcend culture, education, and clinical risk factors. Future studies are necessary to better validate cognitive assessments in low‐education populations, globally.